# Evaluation of semi-quantitative compared to quantitative cultures of tracheal aspirates for the yield of culturable respiratory pathogens – a cross-sectional study

**DOI:** 10.1186/s12890-020-01311-7

**Published:** 2020-10-29

**Authors:** Salima Rattani, Joveria Farooqi, Ghazala Jabeen, Saeeda Chandio, Qaiser Kash, Aijaz Khan, Kauser Jabeen

**Affiliations:** Department of Pathology & Laboratory Medicine, The Aga Khan University, Stadium Road, Karachi, 74800 Pakistan

**Keywords:** Tracheal aspirate, Endotracheal suction aspirate, Quantitative culture, Semi-quantitative culture, Lower respiratory tract infections, Pneumonia, Respiratory pathogens, Pakistan

## Abstract

**Background:**

Diagnosis of lower respiratory tract infections (LRTI) depends on the presence of clinical, radiological and microbiological findings. Endotracheal suction aspirate (ETSA) is the commonest respiratory sample sent for culture from intubated patients. Very few studies have compared quantitative and semi-quantitative processing of ETSA cultures for LRTI diagnosis. We determined the diagnostic accuracy of quantitative and semi-quantitative ETSA culture for LRTI diagnosis, agreement between the quantitative and semi quantitative culture techniques and the yield of respiratory pathogens with both methods.

**Methods:**

This was a cross-sectional study conducted at the Aga Khan University clinical laboratory, Karachi, Pakistan. One hundred and seventy-eight ETSA samples sent for routine bacteriological cultures were processed quantitatively as part of regular specimen processing method and semi-quantitatively. Sensitivity, specificity, positive predictive value (PPV), negative predictive value (NPV) and diagnostic accuracy was calculated for both methods using clinical diagnosis of pneumonia as reference standard. Agreement between the quantitative and semi quantitative methods was assessed via the kappa statistic test. Pathogen yield between the two methods was compared using Pearson’s chi-square test.

**Results:**

The quantitative and semi-quantitative methods yielded pathogens in 81 (45.5%) and 85 (47.8%) cases respectively. There was complete concordance of both techniques in 155 (87.1%) ETSA samples. No growth was observed in 45 (25.3%) ETSA specimens with quantitative culture and 37 (20.8%) cases by semi-quantitative culture. The diagnostic accuracy of both techniques were comparable; 64.6% for quantitative and 64.0% for semi-quantitative culture. The kappa agreement was found to be 0.84 (95% CI, 0.77–0.91) representing almost perfect agreement between the two methods. Although semi-quantitative cultures yielded more pathogens (47.8%) as compared to quantitative ETSA cultures (45.5%), the difference was only 2.3%. However, this difference achieved statistical (chi-square *p*-value < 0.001) favoring semi-quantitative culture methods over quantitative culture techniques for processing ETSA.

**Conclusion:**

In conclusion, there is a strong agreement between the performances of both methods of processing ETSA cultures in terms of accuracy of LRTI diagnosis. Semi-quantitative cultures of ETSA yielded more pathogens as compared to quantitative cultures. Although both techniques were comparable, we recommend processing of ETSA using semi-quantitative technique due to its ease and reduced processing time.

## Background

Lower respiratory tract infections (LRTI) are a cause of increased morbidity and mortality in critically ill patients [1]. It is one of the leading infective causes of intensive care units (ICU) admissions [2, 3]. Its diagnosis is based on the presence of clinical findings along with radiological and microbiological findings [4]. Types of respiratory samples recommended for culture include sputum, endotracheal suction aspirates (ETSA), bronchoalveolar lavage (BAL), protected brush specimens (PBS). Post-physiotherapy ETSA is a much easier sample to collect in intubated patients compared to an invasive BAL sample. These can be obtained simply and cost effectively with less side-effects as compared to BAL and PBS [5]. Thus the commonest respiratory sample from intubated patients with suspected pneumonia or lower respiratory tract infection (LRTI) sent for microbiological analysis is ETSA [3]. For a proper microbiological diagnosis, a true representative sample is necessary as the respiratory tract, endotracheal tubes and tracheostomies are commonly colonized with normal flora [6]. Usual cultivable pathogens causing LRTI are *Streptococcus pneumoniae*, *Haemophilus influenzae*, *Moraxella catarrhalis, Staphylococcus aureus, Klebsiella pneumoniae* and *Pseudomonas aeruginosa* in patients with CAP [7] while *Acinetobacter species, P. aeruginosa, S. aureus* and *K. pneumoniae* are more prevalent in HAP [8]. Recent guidelines published by the Infectious Disease Society of America (IDSA) in 2016 recommend use of noninvasive semi-quantitative cultures in these patients [9]. However, the international European Respiratory Society (ERS), European Society of Intensive Care Medicine (ESICM), the European Society of Clinical Microbiology and Infectious Diseases (ESCMID) and the Latin American Thoracic Association (ALAT) guidelines for the management of hospital-acquired pneumonia and ventilator-associated pneumonia recommend obtaining distal quantitative samples (prior to any antibiotic treatment) in order to reduce antibiotic exposure in stable patients with suspected ventilator associated pneumonia (VAP) and to improve the accuracy of the results; and a lower respiratory tract sample (distal quantitative including BAL and PSB or proximal quantitative or qualitative culture including ETSA) to focus and narrow the initial empiric antibiotic therapy [10]. There have been multiple studies comparing the results of quantitative BAL cultures with ETSA cultures with similar results [9, 11].Very few studies compare quantitative and semi-quantitative processing of ETSA cultures for pathogen yield. We hypothesized that the performance of ETSA culture using quantitative or semi-quantitative technique for LRTI diagnosis is similar.

Processing of cultures quantitatively is more time consuming and costly as compared to semi-quantitative processing of ETSA. The objective of our study was to determine the diagnostic accuracy of quantitative and semi-quantitative ETSA culture for LRTI diagnosis, agreement between the quantitative and semi quantitative culture techniques and the yield of respiratory pathogens with both methods.

## Methods

This was a cross-sectional study conducted at the microbiology section of clinical laboratory of Aga Khan University Hospital – a tertiary care hospital in Karachi, Pakistan as a part of a quality improvement project. ETSA samples received at the AKUH clinical Laboratories for routine bacteriological cultures, selected by systematic sampling, were included in the study. First five ETSA samples received in the microbiology section after 8 am were processed in parallel by the two methods daily Monday through Thursday, from June to September 2017 and then from October to November 2019. We planned to exclude those ETSA samples which were too small in quantity to be processed by both methods. However, we did not encounter any insufficient samples.

Samples were processed both quantitatively as part of regular specimen processing method for ETSA and semi-quantitatively as well. For quantitative cultures, ETSA were digested using an equal amount of sputasol (dithiothreitol) and mixed on a vortex mixer (1:2 dilution). 100 µl (0.1 ml) of the digested specimen was diluted into 9.9 ml of Ringers' solution (1:200 of the original sample). 10 µl (0.01 ml) of the diluted sample was then inoculated on Blood Colistin Nalidixic Acid Agar (BCNA), Chocolate Agar (CHOC) (both incubated in 5% CO2 at 37 °C) and MacConkey Agar (MAC) (incubated in ambient environment at 37 °C) and streaked in quadrants. The quantitative cultures were considered significant at a count of ≥ 10^5^ colony forming units/ml of pathogenic organisms, i.e. ≥ 5 colonies of the same type of organism on a non-selective medium (CHOC agar). For semi-quantitative cultures, samples were examined for purulence and a loopful of the most purulent part was inoculated on CHOC, BCNA and MAC agars and streaked in quadrants. Results were considered significant if there was moderate to heavy growth (colonies growing up to secondary or tertiary streaks) of organisms known to cause lower respiratory tract infection. Final yield was determined after two days of incubation.

Lower Respiratory Tract Infections (LRTI) include bronchitis and bronchiolitis – commonly caused by viruses and atypical bacteria; community-acquired pneumonia (CAP); hospital-acquired pneumonia (HAP) and ventilator-associated pneumonia (VAP); infections of the pleural space; bronchopulmonary infections in patients with cystic fibrosis; and pneumonia in the immunocompromised host [12]. Community Acquired Pneumonia was defined as a new lung infiltrate plus clinical evidence that the infiltrate was of an infectious origin, which included the new onset of fever, purulent sputum, leukocytosis, and decline in oxygenation, excluding hospital acquired pneumonia. Hospital Acquired Pneumonia was defined as a pneumonia not incubating at the time of hospital admission and occurring 48 h or more after admission. Ventilator Associated Pneumonia was defined as pneumonia in mechanically ventilated patients that developed later than or at 48 h after the patient was placed on mechanical ventilation [9].

Concordance between culture methods was expressed as a percentage and was determined by comparing the quantitative and semi-quantitative culture results of ETSA. The results were considered to be completely concordant if both culture methods yielded either no growth or had identical growth of pathogens. The growth of pathogens by ETSA below the cutoff value of ≥ 10^5^ colony forming units/ml or of oral flora such as alpha hemolytic streptococci or yeast was considered as no significant bacterial pathogen isolated. Yield of no organisms on culture after two days of incubation was considered as no growth. The results were considered to be completely discordant when growth of pathogens occurred via one method and not by the other, or when pathogens grew via both methods but the isolates identified were different.

A minimum sample size of 173 ETSA samples to be processed by both quantitative and semi-quantitative culture techniques was calculated to determine with 95% confidence whether pathogen yield is comparable between the two methods.

Statistical analysis was performed using SPSS version 23. Means and standard deviation were used to compute continuous variables like age. Frequency and percentage were used to analyze qualitative variables like gender. Pathogen yield between the two methods was compared using Pearson’s chi-square test. Agreement between the quantitative and semi quantitative microbiological results obtained with ETSA was assessed via the kappa statistic test and interpreted as follows: values ≤ 0 as no agreement, and 0.01–0.20 as none to slight, 0.21–0.40 as fair, 0.41– 0.60 as moderate, 0.61–0.80 as substantial, and 0.81–1.00 as almost perfect agreement [13]. Sensitivity, specificity, positive predictive value (PPV), negative predictive value (NPV), postive likelihood ratio (PLR), negative likelihood ratio (NLR) and diagnostic accuracy was calculated for both methods using clinical diagnosis of LRTI as reference standard.

## Results

One hundred and seventy-eight ETSA samples were processed by both quantitative and semi-quantitative culture techniques. These included samples from patients with HAP including VAP and VAE along with CAP, aspiration pneumonia and bronchopulmonary disease. The general characteristics for the patients are shown in Table 1.

**Table 1:** General characteristics of study population including gender, age distribution, clinical diagnosis and organisms isolated

*Number of samples*	178
*Gender*	
*Male*	122
*Female*	56
*Age*	
* ≤ 1 month*	2
* ≤ 1 year*	11
> *1 year to 5 years*	06
*6–17 years*	2
*18–64 years*	104
* ≥ 65 years*	53
*Lower respiratory tract infection (LRTI)*	96
*Hospital Acquired pneumonia (HAP)*	63
*Ventilator associated pneumonia (VAP)*	48
*Ventilator associated event (including Tracheitis)*	15
*Community acquired pneumonia (CAP)*	4
*Aspiration pneumonia*	11
*Bronchopulmonary Disease (COPD, Asthma, CF)*	7
*Others*	11
*Organisms*	
*Staphylococcus aureus*	18
*Pseudomonas aeruginosa*	19
*Acinetobacter species*	22
*Klebsiella pneumoniae*	20
*Other organisms*	20
*Gram negative bacilli*	
*Escherichia coli*	7
*Stenotrophomonas maltophilia*	5
*Serratia species*	1
*CAP pathogens*	
*Streptococcus pneumoniae*	2
*Haemophilus influenzae*	1
*Molds*	3

**Fig. 1:**
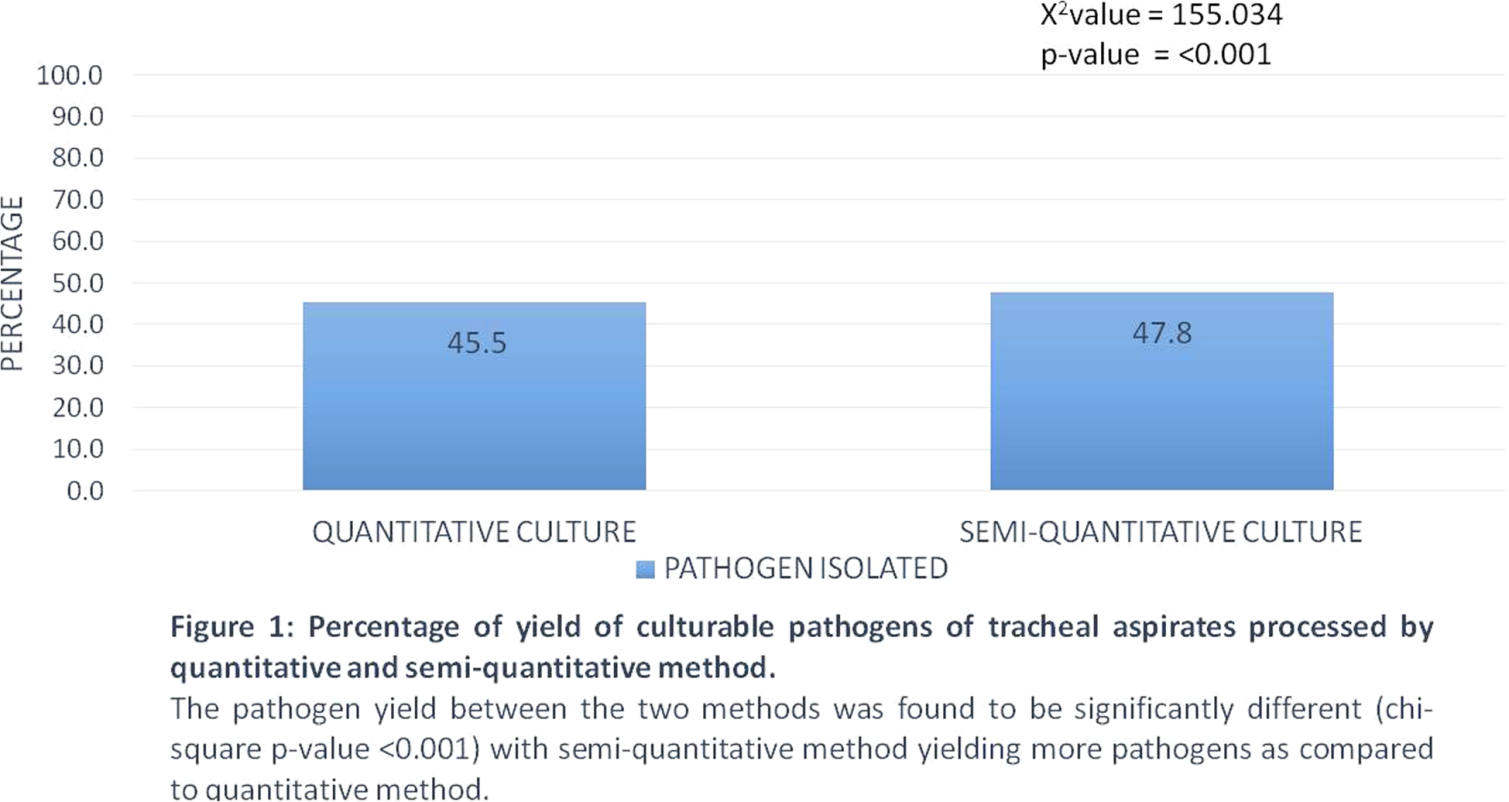
Percentage of yield of culturable pathogens of tracheal aspirates processed by quantitative and semi-quantitative method. The pathogen yield between the two methods was found to be significantly different (chi-square *p*-value < 0.001) with semi-quantitative method yielding more pathogens as compared to quantitative method

The pathogen yield between the two methods was found to be significantly different (chi-square *p*-value < 0.001) with semi-quantitative cultures yielding more pathogens (47.8%) as compared to quantitative ETSA cultures (45.5%). Even though, the difference was only 2.3%, this difference achieved statistical significance favoring semi-quantitative culture methods over quantitative culture techniques for processing ETSA. The quantitative method and semi-quantitative method revealed no growth in 45 (25.3%) and 37 (20.8%) cases, no significant bacterial pathogen was isolated in 52 (29.2%) and 56 (31.5%) cases while pathogens were isolated in 81 (45.5%) and 85 (47.8%) cases respectively, as shown in Fig. 1.

The most common pathogens isolated included *P. aeruginosa, S. aureus, Acinetobacter species, K. pneumoniae*. More than one pathogen was isolated in 20 cases. Table 2 shows the details of pathogens isolated.

**Table 2:** Percentage of isolation of different pathogens by quantitative and semi-quantitative ETSA cultures and by both methods

Organism Isolated	By Quantitative Method n (%)	By Semi-Quantitative Method n (%)	By Both Method n (%)
*Acinetobacter species*	22 (23.2)	23 (23.7)	22 (23.7)
*Klebsiella pneumoniae*	18 (19.0)	20 (21.0)	18 (19.4)
*Pseudomonas aeruginosa*	18 (19.0)	18 (18.6)	17 (18.3)
*Staphylococcus aureus*	18 (19.0)	17 (17.5)	17 (18.3)
*Escherichia coli*	7 (7.4)	7 (7.2)	7 (7.5)
*Stenotrophomonas maltophilia*	5 (5.3)	5 (5.2)	5 (5.4)
*Serratia species*	1 (1.1)	1 (1.0)	1 (1.1)
*Streptococcus pneumoniae*	2 (2.1)	2 (2.1)	2 (2.2)
*Haemophilus influenzae*	1 (1.1)	1 (1.0)	1 (1.1)
Molds	3 (3.2)	3 (3.1)	3 (3.2)
Total organisms isolated	95	97	93

Out of the 178 samples that were processed both quantitatively and semi-quantitatively, there was complete concordance between 155 (87.1%) samples and discordant results were found in 23 (12.9%) samples. Out of these 23 discordant findings, 6 were due to the presence or absence of oral flora on either culture, 4 were due to the presence of additional organism in semi-quantitative in an insignificant count, 3 were due to an additional organism present on the quantitative as compared to semi-quantitative while 10 were due to presence of additional pathogenic organisms on semi-quantitative in comparison with quantitative. The kappa agreement between the two methods was found to be 0.84 (95% CI, 0.77–0.91) representing strong correlation, if they were compared according to categories of “no significant bacteria isolated”, “no growth” or “pathogen isolated” while it was 0.93 (95% CI, 0.88–0.99) representing almost perfect correlation, if comparison was done on the basis of “pathogen isolated” or “no pathogen isolated”. Table 3 shows the sensitivity, specificity, positive predictive value, negative predictive value and diagnostic accuracy for both methods compared to clinical diagnosis of lower respiratory tract infection.

**Table 3:** Performance of quantitative culture and semi-quantitative culture techniques to detect pathogen for microbiological diagnosis compared to the clinical diagnosis of lower respiratory tract infection

Statistic	Quantitative Culture		Semi Quantitative Culture	
	**Value**	**95% CI**	**Value**	**95% CI**
Sensitivity	59.4%	48.9% to 69.3%	60.4%	49.9% to 70.3%
Specificity	70.9%	59.6% to 80.6%	68.4%	56.9% to 78.4%
Positive Likelihood Ratio	2.04	1.39 to 2.99	1.91	1.33 to 2.74
Negative Likelihood Ratio	0.57	0.43 to 0.76	0.58	0.43 to 0.77
Positive Predictive Value	71.3%	62.9% to 78.4%	69.9%	61.8% to 76.9%
Negative Predictive Value	59.0%	52.0% to 65.5%	58.7%	51.6% to 65.5%
Diagnostic Accuracy	64.6%	57.0% to 71.6%	64.0%	56.4% to 71.1%

## Discussion

The diagnosis of pneumonia in patients with lower respiratory tract infections (LRTI) is challenging and involves clinical, radiological and microbiological criteria. To fulfil the microbiological criteria, ETSA are the easiest and rapidly obtainable noninvasive specimens with semi-quantitative results having highest sensitivity but least specificity [9, 14–17]. Less resources and expertise is needed for semi-quantitative processing which can be done rapidly compared to quantitative processing of ETSA [9].

The present study compares culture results for the two methods of processing ETSA suggesting that the results of these cultures processed using quantitative or semi-quantitative methods are comparable, and there is strong agreement between the results of the two methods. Our results are similar to study by Hoshimoto et al. [1], who found a significant correlation between the two culture techniques.

An interesting finding in our study was that the most common pathogen was *Acinetobacter* species, followed by *Klebsiella pneumonia, Pseudomonas aeruginosa* and *Staphylococcus aureus* showing an abundance of pathogens that commonly cause HAP as most of these were isolated from ETSA samples obtained from admitted patients. Studies from our center have shown atypical pathogens are more common in the etiology of pneumonia [18], however, recently, amongst cultivable organisms, *Staphylococcus aureus* was the most common pathogen causing pneumonia, with *S. pneumoniae* and *P. aeruginosa* being close seconds [19]. Another study looking at bacterial etiology of pneumonia in immunocompetent hospitalized patients showed *Pseudomonas aeruginosa* to be the most common causative agent in Pakistan [20]. Only 2 samples were positive for *Streptococcus pneumoniae,* historically considered the most common etiologic agent of CAP worldwide. Patients in Pakistan receive empiric antibiotics in outpatient and inpatient settings without prior microbiologic confirmation of etiologic agents which can lead to false negative cultures, and then later superimposed hospital acquired infections. These may be factors that influenced our low culture rates and a spectrum suggestive of nosocomial etiology [18].

The sensitivity and specificity of semi-quantitative culture for diagnosis of LRTI in our study was 60.4% and 68.3% respectively which is comparable to the study conducted by Fujitani et al. who showed a sensitivity and specificity of semi-quantitative endotracheal aspirate culture to be 65.4% and 56.1% respectively [11]. Multiple studies comparing invasive and non-invasive lower respiratory tract cultures for diagnosis of VAP have been done which show no difference in 28-day mortality, overall mortality, length of ICU stay, duration of mechanical ventilation, or antibiotic changes [5, 6, 8, 9]. The 2016 IDSA guidelines [9] showed a summary of performance characteristics ETSA for microbiological diagnosis of pneumonia. They stated that sensitivity and specificity was 75% (95% CI, 58–88) and 47% (95% CI, 29–65) respectively for ETSA with any amount of growth; positive predictive values ranged from 61% (95% CI, 45–76) for ETSA with any amount of growth to 81% (95% CI, 67%–91%) for ETSA with ≥ 10^5^ CFU/ml. On the basis of these results they recommended noninvasive sampling with semi-quantitative cultures to diagnose VAP, rather than invasive or noninvasive sampling with quantitative cultures which is supported by our study as well.

Based on these recommendations and with our findings supporting the equivalence of the two techniques, laboratories could switch to semi-quantitative processing technique. An additional benefit of semi-quantitative processing for ETSA is reduction of technologist time, reagent consumption and chances for laboratory contamination due to less sample manipulation. This will in turn decrease the overall test cost for ETSA cultures.

There are several important limitations to this study that deserve attention. The sample size was relatively small. This was a single center study and most of the patients were hospitalized which explains the abundance of pathogens commonly associated with HAP, hence it may not be representative of other institutions. However, given that tracheal secretions are often submitted to the microbiology laboratory as part of an empiric work-up for fever in a hospitalized patient, it is suspected that the data here are generally representative of institutions where ETSA culture is routinely performed.

## Conclusion

In conclusion, there is a strong agreement between the performances of both methods of processing ETSA cultures in terms of accuracy of diagnosis of LRTI. Semi-quantitative cultures of ETSA yielded more pathogens as compared to quantitative cultures. Although both techniques were comparable, we recommend processing of ETSA using semi-quantitative technique due to its ease and reduced processing time.

## Data Availability

The datasets used and/or analysed during the current study are available from the corresponding author on reasonable request.

## Abbreviations

CAP: Community acquired pneumonia
HAP: Hospital acquired pneumonia
LRTI: Lower respiratory tract infection
VAP: Ventilator associated pneumonia
ETSA: Endotracheal suction aspirates
BAL: Bronchoalveolar lavage
PBS: Protected brush specimens
ICU: Intensive care unit
IDSA: Infectious Disease Society of America
ERS: European Respiratory Society
ESICM: European Society of Intensive Care Medicine
ESCMID: European Society of Clinical Microbiology Infectious Diseases
ALAT: Latin American Thoracic Association
BCNA: Blood Colistin Nalidixic Acid Agar
CHOC: Chocolate Agar
MAC: MacConkey Agar
PPV: Positive predictive value
NPV: Negative predictive value
